# Supplementary material for: The Systems Biology Research Tool: evolvable open-source software
Source: BMC Syst Biol. 2008 Jun 29;2:55. doi: 10.1186/1752-0509-2-55 (PMC2446383; doi:10.1186/1752-0509-2-55)
Supplement: Additional file 1 — SBRT Archive. An archive of the current version of the Systems Biology Research Tool. [file 1752-0509-2-55-S1.zip › sbrt-1.4.0/doc/developers_guide/api/sbrt/kernel/math/MathExpr.html]

MathExpr


|  |  |  |
| --- | --- | --- |
| **Overview** \| **Package** \| **Class** \| **Use** \| **Tree** \| **Deprecated** \| **Index** \| **Help** | |  |
| **PREV CLASS**   **NEXT CLASS** | **FRAMES**    **NO FRAMES**     **All Classes** |  |
| SUMMARY: NESTED \| FIELD \| CONSTR \| METHOD | DETAIL: FIELD \| CONSTR \| METHOD |  |


---


## sbrt.kernel.math Interface MathExpr<V>

**Type Parameters:**: `V` - the variable type.

**All Known Subinterfaces:**: LinearComb<V>, Polynomial<V>, QuadraticExpr<V>

**All Known Implementing Classes:**: AbstractLinearComb, AbstractPolynomial, HashLinearComb

---

``` public interface MathExpr<V> ```

This interface is used to represent mathematical expressions.

A mathematical expression contains a numerical constant and
a set of variables to which numerical values may be assigned.
This interface captures these basic facts about mathematical
expressions.

**Author:**
:   This interface was written and documented by
    Jeremiah Wright while in the Wagner lab.

---

| **Method Summary** | |
| --- | --- |
| `boolean` | `equals(java.lang.Object obj)`             Indicates if the provided object is equal to this mathematical expression. |
| `double` | `getConstant()`             Returns the numerical constant of this mathematical expression. |
| `java.util.Set<V>` | `getVariables()`             Returns the set of variables contained in this mathematical expression. |
| `boolean` | `hasAllVariables(java.util.Collection<?> variables)`             Indicates if this mathematical expression contains all of the variables in the provided collection. |
| `int` | `hashCode()`             Returns a content-based hash code for this mathematical expression. |
| `boolean` | `hasVariable(java.lang.Object variable)`             Indicates if the provided object is a variable contained in this mathematical expression. |
| `boolean` | `hasVariables()`             Indicates if this mathematical expression contains any variables. |
| `MathExpr<V>` | `plugInNumbers(java.util.Map<?,java.lang.Double> variableValues)`             Plugs in the provided numerical values to produce a new mathematical expression. |
| `java.lang.String` | `toString()`             Returns an appropriate string representation of this mathematical expression. |
| `int` | `variables()`             Returns the number of variables in this mathematical expression. |

**Method Detail**

### getConstant

```
double getConstant()
```

:   Returns the numerical constant of this mathematical expression.

    :   **Returns:**: the numerical constant of this mathematical expression.

---


### plugInNumbers

```
MathExpr<V> plugInNumbers(java.util.Map<?,java.lang.Double> variableValues)
```

:   Plugs in the provided numerical values to produce a new
    mathematical expression.

    If some of the keys in the provided map are not variables
    in this expression, their provided value is simply ignored.
    If a value is provided for each variable in this mathematical
    expression, the resulting expression will not contain any
    variables of its own, only a constant.

    :   **Parameters:**: `variableValues` - objects mapped to numerical values. **Returns:**: the mathematical expression obtained by "plugging in" the provided numerical values.

---


### getVariables

```
java.util.Set<V> getVariables()
```

:   Returns the set of variables contained in this
    mathematical expression.

    :   **Returns:**: the set of variables contained in this mathematical expression.

---


### variables

```
int variables()
```

:   Returns the number of variables in this mathematical
    expression.

    :   **Returns:**: the number of variables in this mathematical expression.

---


### hasVariable

```
boolean hasVariable(java.lang.Object variable)
```

:   Indicates if the provided object is a variable contained
    in this mathematical expression.

    :   **Parameters:**: `variable` - the variable whose presence in this mathematical expression is to be tested. **Returns:**: `true` if this expression contains the provided variable; `false` otherwise.

---


### hasVariables

```
boolean hasVariables()
```

:   Indicates if this mathematical expression contains any
    variables.

    :   **Returns:**: `true` if this expression contains at least one variable; `false` otherwise.

---


### hasAllVariables

```
boolean hasAllVariables(java.util.Collection<?> variables)
```

:   Indicates if this mathematical expression contains all
    of the variables in the provided collection.

    :   **Parameters:**: `variables` - the collection of variables whose presence in this expression is to be tested. **Returns:**: `true` if each element of the provided collection is a variable in this expression; `false` otherwise.

---


### hashCode

```
int hashCode()
```

:   Returns a content-based hash code for this mathematical
    expression.

    :   **Overrides:**: `hashCode` in class `java.lang.Object`
    :   **Returns:**: a content-based hash code for this mathematical expression.

---


### equals

```
boolean equals(java.lang.Object obj)
```

:   Indicates if the provided object is equal to this
    mathematical expression.

    At minimum, for two expressions to be considered equal,
    they must both contain the same variables and constant.
    A stricter definition of equality is left to the
    extenders and implementors of this interface.

    :   **Overrides:**: `equals` in class `java.lang.Object`
    :   **Parameters:**: `obj` - the object to be tested for equality with this mathematical expression. **Returns:**: `true` if this mathematical expression is equal to the provided object; `false` otherwise.

---


### toString

```
java.lang.String toString()
```

:   Returns an appropriate string representation of this
    mathematical expression.

    :   **Overrides:**: `toString` in class `java.lang.Object`
    :   **Returns:**: an appropriate string representation of this mathematical expression.


---


|  |  |  |
| --- | --- | --- |
| **Overview** \| **Package** \| **Class** \| **Use** \| **Tree** \| **Deprecated** \| **Index** \| **Help** | |  |
| **PREV CLASS**   **NEXT CLASS** | **FRAMES**    **NO FRAMES**     **All Classes** |  |
| SUMMARY: NESTED \| FIELD \| CONSTR \| METHOD | DETAIL: FIELD \| CONSTR \| METHOD |  |


---
